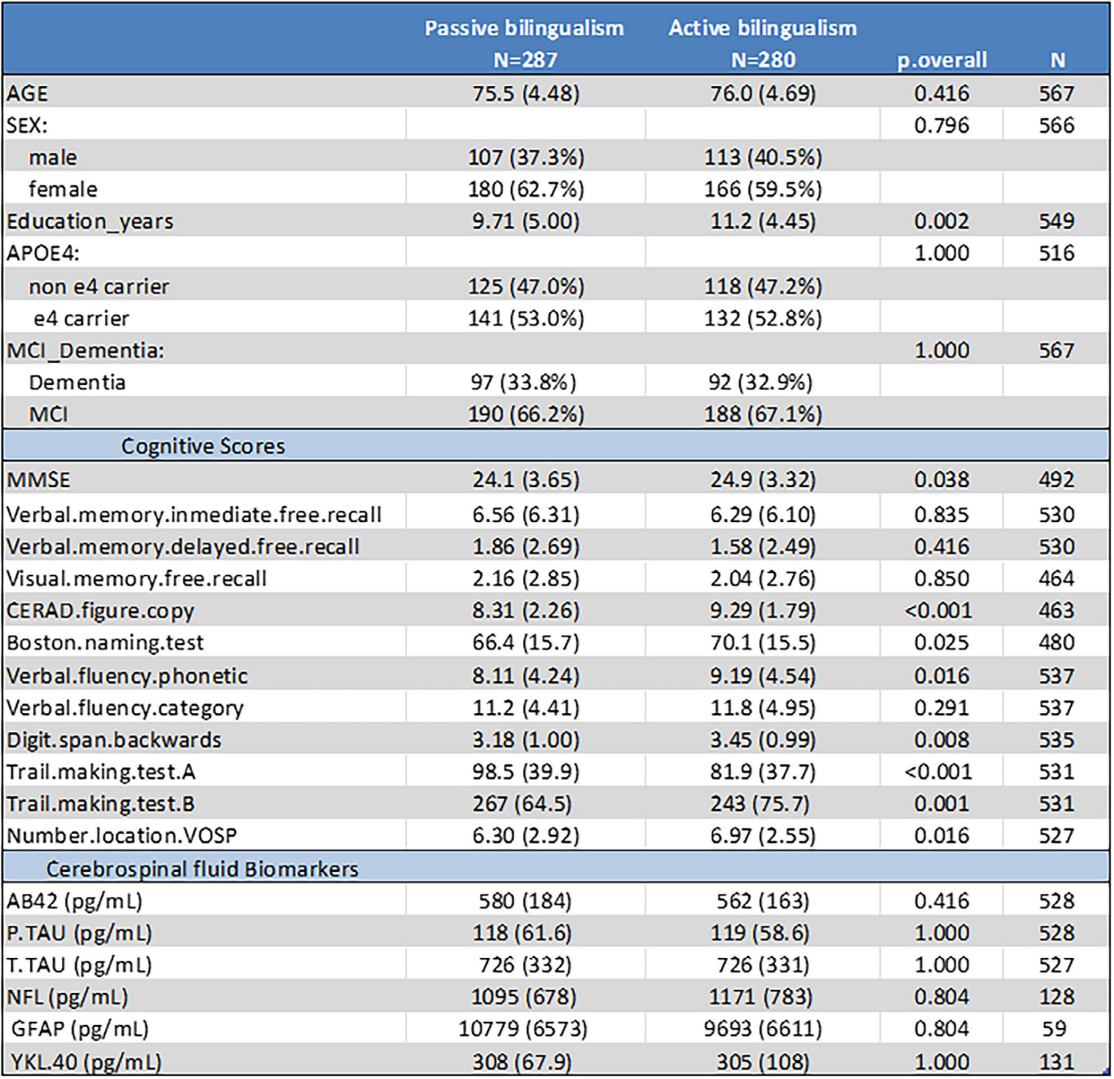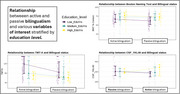# Understanding Bilingualism's Protective Effect in Alzheimer's Disease: Exploring Mechanisms of Resilience vs. Resistance

**DOI:** 10.1002/alz70860_106510

**Published:** 2025-12-23

**Authors:** Miguel A Santos‐Santos, Stephanie M Grasso, Marco Calabria, Isabel Sala, María Belén Sánchez‐Saudinós, Lídia Vaqué‐Alcázar, Alexandre Bejanin, Maria Carmona‐Iragui, Isabel Barroeta, Ignacio Illán‐Gala, Juan Fortea, Olivia Belbin, Daniel Alcolea, Alberto Lleó

**Affiliations:** ^1^ Sant Pau Memory Unit, Hospital de la Santa Creu i Sant Pau ‐ Biomedical Research Institute Sant Pau ‐ Universitat Autònoma de Barcelona, Barcelona, Barcelona, Spain; ^2^ CIBERNED, Network Center for Biomedical Research in Neurodegenerative Diseases, National Institute of Health Carlos III, Madrid, Spain; ^3^ The University of Texas at Austin, Austin, TX, USA; ^4^ Faculty of Health Sciences, Universitat Oberta de Catalunya, Barcelona, Barcelona, Spain; ^5^ Sant Pau Memory Unit, Hospital de la Santa Creu i Sant Pau ‐ Biomedical Research Institute Sant Pau ‐ Universitat Autònoma de Barcelona, Barcelona, Cataluña, Spain; ^6^ Sant Pau Memory Unit, Hospital de la Santa Creu i Sant Pau, Biomedical Research Institute Sant Pau, Universitat Autònoma de Barcelona, Barcelona, Spain; ^7^ Sant Pau Memory Unit, Department of Neurology, Hospital de la Santa Creu i Sant Pau, Institut d'Investigació Biomèdica Sant Pau (IIB SANT PAU), Facultad de Medicina ‐ Universitat Autònoma de Barcelona, Barcelona, Spain; ^8^ Sant Pau Memory Unit, Hospital de la Santa Creu i Sant Pau, Biomedical Research Institute Sant Pau, Universitat Autònoma de Barcelona, Barcelona, Cataluña, Spain; ^9^ Sant Pau Memory Unit, Hospital de la Santa Creu i Sant Pau, Biomedical Research Institute Sant Pau, Barcelona, Spain; ^10^ Hospital de la Santa Creu i Sant Pau ‐ Biomedical Research Institute Sant Pau ‐ Universitat Autònoma de Barcelona, Barcelona, Spain; ^11^ Networking Research Center on Neurodegenerative Diseases (CIBERNED), Instituto de Salud Carlos III, Madrid, Spain

## Abstract

**Background:**

Bilingualism has been proposed as a cognitively enriching factor that delays dementia onset by an average of four years. Understanding how bilingualism may drive variation in cognitive decline—whether by conferring resilience or resistance to Alzheimer's disease (AD) pathology—is critical for its therapeutic potential. Approaching the issue from a biological perspective is essential, however few studies have examined its underlying mechanisms. This study investigates the relationship between bilingual status, cognitive decline, and cerebrospinal fluid (CSF) biomarkers reflecting key AD pathophysiological processes.

**Method:**

We analyzed data from 567 participants with biomarker‐confirmed, memory‐predominant AD in the Sant Pau Initiative on Neurodegeneration (SPIN) cohort. All participants underwent neurologic and neuropsychological evaluations, CSF biomarker testing, and language‐use assessments. They were categorized into active bilinguals (frequent Catalan and Spanish use) and passive bilinguals (Spanish speakers with passive Catalan exposure). Robust and generalized linear models were used to determine whether bilingualism protects against cognitive decline and, if so, whether this reflects a mechanism consistent with resilience (better cognitive performance despite similar levels of pathology) or resistance (lower CSF biomarker levels reflecting amyloid‐beta (Aβ42) and tau accumulation (phospho‐tau, total‐tau), neurodegeneration (neurofilament light, NFL), and neuroinflammation (GFAP, YKL‐40).

**Result:**

Active and passive bilinguals were similar in age, sex, APOE genotype distribution, and proportion of participants in the MCI/dementia stage, though active bilinguals had more years of education (Table 1). Controlling for education, its interaction with bilingual status, and age, we found evidence of resilience: active bilingualism was consistently associated with better performance on the Trail Making Test A, CERAD figure copy, and Boston Naming Test. Evidence of resistance was more limited: active bilingualism was linked to lower levels of YKL‐40. This effect was present only in low‐ and mid‐education participants, as evidenced by the significant interaction between bilingual status and education. No significant associations were found with the other biomarkers.

**Conclusion:**

Active bilingualism appears to protect against cognitive decline in individuals with biomarker‐confirmed AD, primarily through resilience rather than resistance to AD pathology. Preliminary findings suggest a potential effect of resistance to neuroinflammation is limited to people with AD with low and mid‐education levels, which requires further investigation in larger datasets.